# A second generation framework for the analysis of microsatellites in expressed sequence tags and the development of EST-SSR markers for a conifer, *Cryptomeria japonica*

**DOI:** 10.1186/1471-2164-13-136

**Published:** 2012-04-16

**Authors:** Saneyoshi Ueno, Yoshinari Moriguchi, Kentaro Uchiyama, Tokuko Ujino-Ihara, Norihiro Futamura, Tetsuya Sakurai, Kenji Shinohara, Yoshihiko Tsumura

**Affiliations:** 1 Department of Forest Genetics, Forestry and Forest Products Research Institute, 1 Matsunosato, Tsukuba, Ibaraki, 305-8687, Japan; 2 Department of Molecular and Cell Biology, Forestry and Forest Products Research Institute, 1 Matsunosato, Tsukuba, Ibaraki, 305-8687, Japan; 3 Integrated Genome Informatics Research Unit, Plant Science Center, RIKEN Yokohama Institute, 1-7-22 Suehiro-cho, Tsurumi-ku, Yokohama City, Kanagawa, 230-0045, Japan

## Abstract

**Background:**

Microsatellites or simple sequence repeats (SSRs) in expressed sequence tags (ESTs) are useful resources for genome analysis because of their abundance, functionality and polymorphism. The advent of commercial second generation sequencing machines has lead to new strategies for developing EST-SSR markers, necessitating the development of bioinformatic framework that can keep pace with the increasing quality and quantity of sequence data produced. We describe an open scheme for analyzing ESTs and developing EST-SSR markers from reads collected by Sanger sequencing and pyrosequencing of sugi (*Cryptomeria japonica*).

**Results:**

We collected 141,097 sequence reads by Sanger sequencing and 1,333,444 by pyrosequencing. After trimming contaminant and low quality sequences, 118,319 Sanger and 1,201,150 pyrosequencing reads were passed to the MIRA assembler, generating 81,284 contigs that were analysed for SSRs. 4,059 SSRs were found in 3,694 (4.54%) contigs, giving an SSR frequency lower than that in seven other plant species with gene indices (5.4–21.9%). The average GC content of the SSR-containing contigs was 41.55%, compared to 40.23% for all contigs. Tri-SSRs were the most common SSRs; the most common motif was AT, which was found in 655 (46.3%) di-SSRs, followed by the AAG motif, found in 342 (25.9%) tri-SSRs. Most (72.8%) tri-SSRs were in coding regions, but 55.6% of the di-SSRs were in non-coding regions; the AT motif was most abundant in 3′ untranslated regions. Gene ontology (GO) annotations showed that six GO terms were significantly overrepresented within SSR-containing contigs. Forty–four EST-SSR markers were developed from 192 primer pairs using two pipelines: read2Marker and the newly-developed CMiB, which combines several open tools. Markers resulting from both pipelines showed no differences in PCR success rate and polymorphisms, but PCR success and polymorphism were significantly affected by the expected PCR product size and number of SSR repeats, respectively. EST-SSR markers exhibited less polymorphism than genomic SSRs.

**Conclusions:**

We have created a new open pipeline for developing EST-SSR markers and applied it in a comprehensive analysis of EST-SSRs and EST-SSR markers in *C. japonica*. The results will be useful in genomic analyses of conifers and other non-model species.

## Background

With the advent of the second generation sequencing technologies [1], more and more sequences are being generated on a daily basis from various organisms in order to analyze genome sequences, whole transcriptomes, molecular evolution, and metagenomes (reviewed in [2-7]). The availability of genome and/or reference sequence data for a specific organism greatly facilitates the study of DNA-protein interactions, epigenetics and single nucleotide polymorphisms (SNPs). Second generation sequencing has thus triggered a revolution in many areas of biology. While there are many non-model organisms whose genomes have not yet been fully sequenced, the decreasing costs of sequencing have prompted qualitative changes in research strategies, including those relating to the development of molecular markers (which are indispensible for breeding studies) and diagnostics in the agricultural and medical sciences.

Microsatellites, or simple sequence repeats (SSRs), are tandem repeat sequences with a repeating unit of one to six base pairs that are abundant in most genomes and exhibit high levels of polymorphism. They are therefore useful molecular markers, although the process of developing SSR markers originally involved laborious screening, cloning and sequencing steps [8]. Together with increases in the throughput of commercial sequencers, the availability of a large body of data demonstrating that SSRs are abundant in expressed sequence tags (ESTs) [9], has made it viable to develop EST-SSR markers using traditional Sanger sequencing. In recent years, the use of second generation sequencing machines has become increasingly common in EST-SSR marker development [10-13]. The longer reads produced by the Roche Genome Sequencer using the pyrosequencing method are preferred for this purpose, especially when working with non-model organisms for which comparatively few genomic resources and reference sequences are available. In such cases, the ability to sequence longer fragments increases the uniqueness of the sequences obtained, reducing the computational challenges associated with their analysis.

There are several automated pipelines for cleaning up and assembling reads, detecting SSRs, and designing primers that target the detected SSRs [14-16]. These pipelines are integrated systems; as such, in the absence of expert knowledge, they are not readily customized to meet any additional needs the user might have. To address this issue and to facilitate the ongoing development of analytical software at a pace that matches the rapid evolution of sequencing systems, it would be beneficial to create modular pipeline systems for the analysis of SSRs in ESTs and the development of EST-SSR markers. Such modular systems would be more flexible than current solutions and could be readily upgraded or adjusted in response to new developments in sequencing technology.

In this paper, we explicitly describe a framework for analysing EST-SSRs and designing primers to target them using transcriptome shotgun assembly (TSA), on the basis of reads collected using the Sanger and pyrosequencing methods. The model organism used was sugi (*Cryptomeria japonica* D. Don), Japanese cedar, which is an industrially important coniferous species growing in Japan. We have previously collected ESTs (some of which are full-length and enriched) from several tissue libraries for this species [17-20], used them to develop cleaved amplified polymorphic sequence (CAPS) [21-23], SSR [24,25] and EST-SSR [26] markers, and constructed genetic linkage maps [27]. The advantage of using *C. japonica* in genomic studies rather than other conifer species is that the estimated C-value for *C. japonica* is 11 pg [28], meaning that its genome is approximately half the size of those of *Pinus* or *Picea*[29,30]. While *Cryptomeria* is one of the Cupressease, *Pinus* and *Picea* belong to the Pinaceae. The gap between these two families is the deepest phylogeneitc split in the conifers [31], and may affect genomic composition of both family members. Features identified by analyzing EST-SSR can be referenced to study phylogenetic relationships among conifers. In the present study, TSA was performed using around 118 k Sanger and 1.2 M pyrosequencing reads, resulting in 81,284 contigs that served as an important central hub for downstream analysis. The characteristics of the SSRs in the ESTs were analyzed in terms of frequency, GC percent, location, and gene ontology. We then used gene indices to compare the results obtained for *C. japonica* to those observed in other plant species. Comparative analysis was used to identify the distinguishing features of this species, showing that much less amount of AT motif in *C. japonica* than in two Pinaceae species. EST-SSR markers were developed using an open pipeline, and factors affecting PCR success and the level of polymorphisms were analyzed using a generalized linear model. This is the first comprehensive analysis of SSR-containing ESTs and EST-SSR markers in *C. japonica*; the results obtained will be useful in future genomic analyses of conifers and other non-model species.

## Methods

A web link to programs/resources that were used in the present study is provided in Additional file 1: Table S1.

### cDNA sequencing

Sanger sequencing was used to collect 141,097 reads for unigene construction. These reads had been obtained in previous studies (Table 1). In the course of this work, two new cDNA libraries (CFFL and CLFL, full length enriched; Futamura et al. in preparation) were sequenced and subjected to assembly. A cDNA library for pyrosequencing was constructed from 10 seedlings from 10 elite *C. japonica* trees in Japan. Total RNA was extracted from each seedling using a CTAB-based method [32] and the extracted RNA from each individual was mixed in an equimolar fashion. cDNA synthesis was carried out using the SMART cDNA construction kit (Clontech) and normalized using a cDNA Normalization Kit (Evrogen) by the Dragon Genomics Centre, TAKARA BIO Inc. (Yokkaichi, Japan). The library was pyrosequenced using Roche 454 GS FLX Titanium reagents in one and a quarter pico-titer plates. The DRA (DDBJ Read Archive) accession number for this project is [DDBJ:DRA000446] and can be accessed at http://trace.ddbj.nig.ac.jp/dra/index_e.shtml. Chimeric reads of the 454-reads were pre-filtered by Dragon Genomics Center. To evaluate read quality, the read length with phred quality ≥ 20 was estimated by measuring the read length after trimming by the qualityTrimmer module of the Euler-SR package [33].

**Table 1:** Sequencing, masking and trimming statistics for unigene assembly

	**Sequencing method**							
	**Sanger**							**Pyrosequencing**
**Library**	**CMFL**	**CFFL**	**CLFL**	**CM**	**CF**	**CC**	**Total**	**TUM25**
Number of reads (1)	39936	N/A	N/A	3456	768	5184	129195	1333444
Number of 3' ESTs in (1)	19968	N/A	N/A	2880	0	0	62776	-
Number of base call (amount of sequences in Mbp) for (1)	30.12	N/A	N/A	2.97	0.53	4.95	141.66	393.8
Amount of bases (Mbp) masked after cross_match	4.07	N/A	N/A	0.16	0.16	0.59	14.04	0.09
Number of reads after SeqClean (reads passed to assembly) (2)	36722	N/A	N/A	3175	485	4435	118319	1201150
Amount of reads (Mbp) after SeqClean (reads passed to assembly)	19.95	N/A	N/A	1.96	0.23	2.26	78.59	354.5
Aveage read length (bp) in (2) with phred QV > =20	535.8	N/A	N/A	609.3	460.2	500.5	654.2	282.7
Tissue/developmental stage	Male bud	Female bud	Leaf	Male flower	Female flower	Inner bark	–	Seedling
References	[17]	Futamura et al. in prep.	Futamura et al. in prep.	[19]	[19]	[20]	–	This study

N/A: See Futamura et al. in preparation for details.

### Construction of unigene elements

Electropherograms were base-called using the phred program [34,35]. All Sanger reads (141,097 sequences) were screened by cross_match [36] for vectors (with the parameters:-minmatch 10 -minscore 19), adaptors (with the parameters:-minmatch 10 -minscore 12) and the genome sequence of *Escherichia coli* (with the parameters:-minmatch 20 -minscore 30). For 454-reads, adaptors were screened and masked with cross_match, using the parameters:-minmatch 10 -minscore 17. SeqClean [37] was also used to screen all Sanger reads for vector, adaptor and *E. coli* genomic sequences and all 454-reads for adaptors and chloroplast sequences [38]; default parameters were used in this case, and sequences shorter than 100 bp were considered invalid. Finally, the longest non-masked region was extracted using an in-house perl script to eliminate potential chimeras. This process yielded 118,319 Sanger reads and 1,201,150 pyrosequence reads. MIRA (3.2.0) [39] was used to directly assemble the Sanger and pyrosequence reads, with the standard options (−job = denovo,est,accurate,sanger,454) and no supplementary XML files. MIRA was also used for all assemblies conducted during this study. The GC percentage of the contigs was calculated using an in-house perl script.

### Mining of microsatellites

The MISA [40] software package was used to analyze microsatellite (SSR) frequencies. The minimum numbers of repeats for SSR detection were as follows: six for di-SSRs, five for tri-SSRs, four for tetra-SSRs, three for penta-SSRs and three for hexa-SSRs. The maximum length of interruption between two adjacent SSR repeat units was set to zero bp. The same criteria were used for all analyses of SSR frequency. SSR frequencies were analyzed for 81,284 *C. japonica* contigs (CjCon1) and seven gene indices [41] in order to compare SSR frequencies between taxa. SSR frequencies were also calculated for each cDNA library to identify frequency differences between tissue/stage types and between sequencing directions (i.e. between 5′ and 3′ ESTs). Reads from each library or sequencing group were assembled using MIRA (3.2.0) with parameters appropriate for the type of sequencing used (either -job = denovo, est, accurate, sanger or -job = denovo,est,accurate,454). We defined five tissue or stage types according to the origin of the cDNA (bark, leaf, female bud, male bud and seedling). For bark tissue, 11,611 ESTs [18,20] from the cambium and surrounding tissues (inner bark) were retrieved from dbEST, with 3,114 and 6,273 reads being identified as 3′ and 5′ ESTs, respectively. For ESTs from other tissues and sequencing directions, the libraries listed in Table 1 were used (CLFL for leaves, CMFL for male buds and CFFL for female buds).

To compare the frequency of EST-SSRs in *C. japonica* with that in other species, seven gene indices were downloaded and analyzed using MISA with the parameters listed above. We used the following TIGR gene indices [41]: AGI (Arabidopsis_thaliana release_15), HAGI (Helianthus_annuus release_6), NTGI (Nicotiana_tabacum release_6), OSGI (Oryza_sativa release_19), OGI (Oak release_2), SGI (Picea release_4) and PGI (Pine release_9), which were available from http://compbio.dfci.harvard.edu/tgi/. These gene indices were selected so as to represent specific phylogenetic classes of land plants: gymnosperms (SGI and PGI), monocots (OSGI), rosid I (OGI), rosid II (AGI), asterid I (NTGI) and asterid II (HAGI). The relationship between genome size and the frequency of EST-SSRs was analyzed using data from the Plant DNA C-values Database [42,43].

The location of SSRs within contigs was estimated using prot4EST (ver. 3.1b) [44]. This program uses hierarchical steps to identify protein coding regions. ESTScan [45] was used in the second step of the process, with a matrix file constructed from 3,644 representative peptide sequences that were estimated using FrameDP [46] with the default parameters and the CjCon1; the TAIR9_pep sequences (available at ftp://ftp.arabidopsis.org/home/tair/Sequences/blast_datasets/) were used as reference material. These representative peptides were generated from 4,222 full length cDNA candidates and were clustered using BlastCLUST, a component of the BLAST package [47], with the following parameters: -p T -b F -L 0.5 -S 60. While FrameDP alone can be used to estimate coding regions, preliminary analysis of the predicted SSR locations (coding or non-coding) showed that it over-predicted the presence of SSRs in the coding regions of the 5′ UTR in popular gene models [48]. We therefore chose to rely on hierarchical analyses performed using prot4EST for predicting SSR locations. Predicted peptide sequences were used to estimate the coordinates of coding regions by alignment against corresponding DNA sequences using the fasty35 module of the FASTA package [49].

Functional annotations for SSR-containing contigs were identified on the basis of BLAST [47] similarity searches against the NCBI nr protein database using an evalue cutoff of 1e-3. The BLAST results were related to Gene Ontologies for plants (plant GO slims) using Blast2GO software [50-52]. The enrichment of GO terms for contigs with SSRs was tested using FatiGO [53] through Blast2GO.

### Design and selection of EST-SSR primers

Sequences for primer design were collected from three sources: (1) sequences already registered in dbEST (GroupA); (2) sequences that had not been used for assembly (11,831 reads) (GroupB) and (3) sequences that had been used for assembly (81,284 contigs and 92,541 debris) (GroupC). When the *C. japonica* sequences were downloaded from dbEST (February 1^st^, 2011), we found 56,645 sequences in the database, most of which had been registered by our group.

For GroupA, SeqClean was used with the default parameters to detect contaminant sequences using the UniVec database [54], because dbEST often contains such contaminants [55]. SeqClean was also used to exclude chloroplast sequences of *C. japonica*[38] from GroupA. For GroupB, cross_match was used to mask vector and adaptor sequences, with the parameter set listed above (see the section on “Construction of unigene elements”). The genomic sequence of *E. coli* was also masked using cross_match (with the parameters: -minmatch 100 -minscore 150). In addition, GroupB was screened for vector/adapter and chloroplast sequences using SeqClean with default parameters. For GroupC, low quality regions were removed prior to primer design using the qualityTrimmer program of the Euler-SR package [33], which removed 2.18 Mb (2.36%) of low quality data.

Sequences with SSRs were initially extracted from these three source sequences. 8,166 SSR-containing sequences were identified and passed to downstream processes. Two different pipelines for developing EST-SSR markers were used. The first involved read2Marker scripts [15] that cluster sequences on the basis of their BLAST similarity; primers were designed using Primer3 [56], and the designed primers were further checked for possible mis-annealing during PCR by searching for partial sequence identity within the primer pairs and all template sequences (a set of scripts are available from https://ml-wiki.sys.affrc.go.jp/engei_marker/_media/read2marker_distribution.zip). We used the default parameters for all processes except for those involving Primer3 ( Additional file 2: Table S2).

The other pipeline (CMiB) was newly developed and employs a combination of **C**D-HIT-EST, **M**ISA, **i**pcress and **B**lastCLUST (a typical shell script can be found in Additional file 3: cmib.sh). The initial step involves clustering the SSR-containing sequences using CD-HIT-EST [57] with the following parameters: -c 0.8 -n 4 -r 1 and recovering the longest sequence within each cluster. From the resulting 4,067 unique sequences, primers were designed using the MISA [40] package (misa.pl, p3_in.pl and p3_out.pl scripts) with the same SSR detection criteria as outlined previously except that the length of interruption between two adjacent SSR was set at 100 bp. Primers were designed using Primer3 [56], which was called by the p3_in.pl script (see Additional file 2: Table S2 for a detailed parameter set for primer design). The designed primers were then used for *in silico* PCR experiments using the ipcress command of the exonerate package [58] with the default options. This was applied to the 4,067 unique sequences to select primer pairs that would produce single products. It was necessary to include this step in order to avoid having SSRs on repetitive domains within a single sequence, which are difficult to exclude using between-sequence comparisons alone. Second generation sequencing techniques produce long contigs that necessitate self-sequence comparison. The *in silico* PCR products were further clustered using BlastCLUST, a part of the BLAST package [47], with the following parameters:-p F -b F -L 0.5 -S 90. Finally, the primer pairs that generated the shortest *in silico* product from each cluster were selected. The successful sequences were BLASTed against EST-SSR sequences for which primers had already been designed [24,26]. Sequences with HSP (High-scoring Segment Pairs) scores above 50 were excluded from further analysis.

### PCR and statistical analysis of EST-SSRs

For primer pairs resulting from the read2Marker pipeline, 96 of the 111 primer pairs were selected in an arbitrary and random fashion. For those resulting from the CMiB pipeline, 96 of the 2,371 primer pairs that showed no similarity with previously-reported EST-SSR markers [24,26] were selected at random after the exclusion of primer pairs that had already been selected for the read2Marker pipeline. In total, 192 primer pairs (96 each from read2Marker and CMiB) were synthesized (Operon Biotechnologies, Tokyo Japan). PCR was first carried out for two individuals in 10 μL reaction mixtures containing ca. 5 ng genomic DNA, 1 × PCR buffer, 200 μM of each dNTP, 1.5 mM MgCl_2_, 0.2 μM of each synthesized primer, and 0.25 U of *Taq* polymerase (Promega Madison, USA), using the following program: 94°C for 5 min, then 40 cycles of 94°C for 30 s, 55–62°C for 30 s and 72°C for 30 s, followed by a final extension at 72°C for 5 min. The PCR products were electrophoretically separated on 2% agarose gels and stained with ethidium bromide to check for successful amplification. The utility of EST-SSR primers that produced visible bands on the agarose gel was demonstrated by analyzing polymorphisms among 16 individuals of *C. japonica* from various locations across Japan ( Additional file 4: Figure S1). PCR was carried out in 10 μL reaction mixtures under the conditions described above using the annealing temperatures listed in Additional file 5: Table S3. PCR products were labelled with ChromaTide Rhodamine Green-5-dUTP (Molecular Probes Eugene, USA) according to a method described elsewhere [59], and analyzed using a 3100 Genetic Analyzer with GeneScan software (Applied Biosystems, Foster City, USA). For each locus, the number of alleles (*Na*) was counted and the observed (*H*_*O*_) and expected (*H*_*E*_) heterozygosity was calculated. Polymorphism information content (*PIC*) [60] was calculated using the Excel Microsatellite Toolkit [61]. Deviation from Hardy-Weinberg equilibrium was tested using GenepopV4 [62].

To analyze factors affecting successful PCR amplification, we fitted a generalized linear model with a binomial error distribution, the logit-link function and the PCR amplification as the binary dependent variable (coded as 1 for success and 0 for failure). The explanatory variables were (1) the pipeline used to design the primers (read2Marker or CMiB), (2) estimated primer location (for both forward and reverse primers, in terms of location within or outside an estimated coding region), (3) the sum of the melting temperatures of the forward and reverse primers and (4) the expected PCR product length in base pairs. The primer location was estimated in the same way as for SSRs, as described in the “Mining of microsatellites” section above. We also analyzed the relationships between the level of polymorphism for each locus and factors that might affect it by fitting a generalized linear model with a Poisson error distribution, log-link function and the number of alleles (*Na*) as the dependent variable. The explanatory variables were (1) the pipeline used to design the primers (read2Marker or CMiB), (2) estimated SSR location (within or outside a coding region), (3) maximum number of SSR repeats within the amplified region, and (4) the length of the SSR repeat unit (i.e. di-, tri-, tetra-, penta- or hexa-SSR) corresponding to the maximum repeat within the expected PCR products. Ninety-four loci that were analyzed by capillary electrophoresis were included in this analysis, with *Na* = 1 for monomorphic loci.

We used R 2.11.1 (R Development Core Team 2011) [63] to fit the generalized linear models. The significance of each coefficient in the model was tested using Wald statistics (*z* value).

## Results and discussion

**Figure 1:**
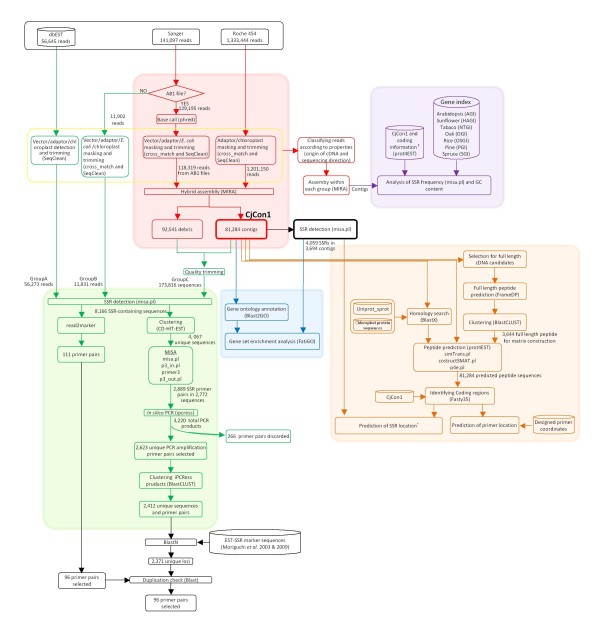
**Schematic representation of the bioinformatic analysis.** Different colours correspond to different kinds of analysis (cleaning in yellow, assembly in red, comparative analysis in purple, location (UTR or coding) analysis based on peptide prediction in orange, gene ontology based analysis in blue and EST-SSR primer design in green). The † mark indicates a logical link between the estimated SSR location and comparative analysis.

Figure 1 shows an analysis scheme for the work reported in this paper. The scheme is divided into 5 sections, each of which is shown in a different colour and corresponds to a specific step in the analytical process; cleaning is shown in yellow, assembly in red, comparative analysis in purple, location (UTR or coding) analysis on the basis of peptide prediction in orange, gene ontology-based analysis in blue, and EST-SSR primer design in green.

### EST cleaning and assembly

In total, 141,097 Sanger sequence reads were considered, although no quality data were available for 11,902 of them; those without quality data were excluded from the assembly. The remaining sequences (129,195) were base-called to produce sequence data covering 141.66 Mbp (Table 1). After cleaning using cross_match [36] and SeqClean [37] (see the Methods section for more detail), 118,319 reads (78.59Mbp) were passed to assembly, with an average read length of 654.2 bp for bases with a phred QV of 20 or above. By using pyrosequencing with Roche GS FLX Titanium reagents, we obtained 1,333,444 reads (393.8 Mbp) after chimera filtering. After cleaning, this number was reduced to 1,201,150 reads (354.5 Mbp) that were passed to assembly, with an average length of 282.7 bp for bases with QV ≥ 20. The length of the sequences masked by cross_match was 14.04 Mbp and 0.09 Mbp for the Sanger and pyrosequencing methods, respectively, which corresponded to 17.9% and 0.025% of the total sequenced length submitted for assembly. Because Sanger sequencing involves a cloning step using adaptors/vectors and host organisms, the sequences obtained using this method include a greater quantity of data that is unnecessary for assembly compared to those obtained by pyrosequencing. While adapters were used for pyrosequencing, the level of contamination was low.

118,319 Sanger and 1,201,150 pyrosequencing reads were assembled using the MIRA program, which identified 81,284 contigs and 92,541 debris reads. These contigs are collectively referred to as CjCon1; in total, they covered 63.57 Mbp. A plot of the length distribution of the reads can be seen in Additional file 6: Figure S2; the average read lengths of 654.2 bp and 282.7 bp obtained by the Sanger method and pyrosequencing, respectively, are highlighted (Table 1). The average length and depth (number of reads assembled in a contig) of CjCon1 was 782.1 bp and 15.1, respectively ( Additional file 7: Figure S3). The most common number of ESTs in a given contig was two; 23,382 (28.8%) of the 81,284 contigs were in this category. 67,114 (82.6%) of the contigs contained fewer than 21 ESTs. The longest contig was 5,049 bp in length and exhibited sequence homology with peroxidase 12; the deepest contig had 1,795 reads exhibited homology with unknown proteins from *Picea* species.

### Assembly within individual libraries and sequencing directions

In order to assess variation, and thus potential bias, in the depth and number of reads across tissues, different EST data sets were constructed for bark, bud, leaf and seedlings. For the bark library, 11,611 sequences were collected from the dbEST database; the sources of the other libraries analyzed were as indicated in Table 1 (i.e. the male bud library was sourced from CMFL, the female bud library from CFFL, the leaf library from CLFL, and the seedling library from TUM25). Assembly using the MIRA program produced contigs with varying average depths. Contigs from the seedling library (TUM25), which was sequenced by the pyrosequencing method, had the greatest average depth (14.9); contigs from the other libraries, which were sequenced by the Sanger method, had average depths ranging from 3.06 to 3.39 (Additional file 8: Table S4). In contrast, the average contig length for the Sanger libraries (692.8 - 999.9 bp) was greater than that for the pyrosequencing library (697.2 bp). The sequences in each library were divided into separate groups on the basis of their sequencing direction (3′ or 5′ ESTs) where sequencing direction data were available. It was found that the average contig depths for 3′ and 5′ assembly were almost identical (2.77 - 3.96), but the average contig length was greater for 5′ assembly (Additional file 8: Table S4). Because 3′ ESTs have a poly-A sequence at their beginning, their sequencing chromatograms are of lower quality than their 5′ counterparts, and 3′ ESTs are generally shorter than 5′ ESTs.

### Frequency of EST-SSRs

SSR motifs and the frequency with which they occur differ greatly between taxa [64,65]. We therefore analyzed both of these factors for *C. japonica* using CjCon1. We also sought to identify differences in SSR frequency that may have arisen from the methods used for library preparation (i.e. the tissues sampled and sequencing direction). Information of this kind is important when designing efficient strategies for developing SSR markers and selecting target SSR motifs.

**Figure 2:**
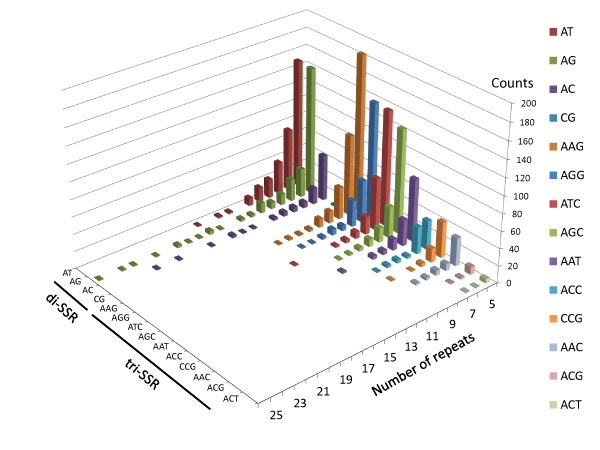
Frequency distribution of SSRs by motif and repeat length in CjCon1.

We used MISA [40] to screen for SSRs within CjCon1 and detected 655, 1,319, 194, 741 and 1,094 di-, tri-, tetra-, penta- and hexa-SSRs, respectively, giving a total of 4,003 pure SSRs. In addition, 56 compound SSRs (i.e. sequences of multiple different contiguous SSRs), were found, giving a grand total of 4,059 SSRs in 3,694 (4.54%) contigs. Most (91.7%) of these contigs contained only one SSR; the greatest number of SSRs detected in a single contig was seven. The most frequent SSR motifs were AT (303 or 46.3% in di-SSRs) and AAG (342 or 25.9% in tri-SSRs) (Table 2). The frequency distributions for each motif and number of repeats are shown in Figure 2; it is apparent that the di-SSRs tended to be the longest. The longest number of repeats was observed in AG motif, (AG)_26_. CjCon1 contained a total of 63.57 Mbp, giving an SSR density of 6.39 SSRs/10 kb.

**Table 2:** SSR motifs and their frequency in 3′ UTR, 5′ UTR and coding regions

		**Estimated location**				
**SSR**	**motif**	**3' UTR**	**5' UTR**	**coding**	**undetermined**	**Total**
di	AT	154	70	73	6	303
	AG	42	53	155	6	256
	AC	27	18	46	3	94
	CG	0	0	2	0	2
	sub-total	223	141	276	15	655
tri	AAG	19	36	285	2	342
	ATG	19	32	178	1	230
	AGG	7	49	170	0	226
	AGC	18	34	129	2	183
	AAT	55	22	49	2	128
	ACC	6	7	64	0	77
	GGC	9	15	38	0	62
	AAC	11	9	32	0	52
	ACG	0	1	11	0	12
	AGT	2	1	4	0	7
	sub-total	146	206	960	7	1319
tetra		61	65	60	8	194
penta		180	193	349	19	741
hexa		154	225	703	12	1094
compound		15	5	35	1	56
Total		779	835	2383	62	4059

SSR location was estimated by inferring coding regions using the prot4EST pipeline [44] and the fasty35 module of the FASTA package [49]. Some of the locations were un-determined because the corresponding SSRs extended over both coding and non-coding regions.

**Figure 3:**
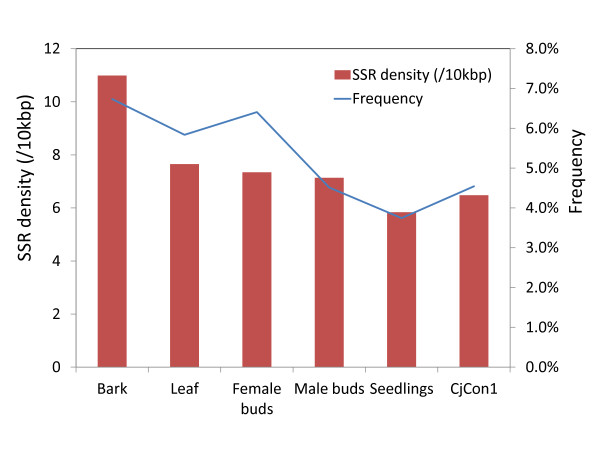
**SSR frequency and density (/kbp) within each library.** SSR frequency was defined as the percentage of SSR containing sequences within contigs, while SSR density was calculated as the number of SSRs in 10 kbp of contigs.

In order to compare the SSR frequency and density of different libraries and sequencing directions, the contigs discussed in the previous section were analysed using MISA [40]. The frequency and density of SSRs for each library ranged from 3.7% to 6.7% and 5.8 to 10.9/10kbp, respectively; the bark library contained the greatest number of SSRs (Figure 3). This may be due to the small number of contigs from the bark library that were screened for SSRs. An analysis of SSR frequency/density as a function of sequencing direction indicated that the SSR frequency/density was higher for 5′ EST assembly than for 3′ assembly (Additional file 9: Figure S4). It may thus be advantageous to sequence from the 5′ direction if there is a need to maximise the number of detected microsatellite sequences when developing microsatellite markers for *C. japonica*. While it was reported that 3′ ESTs contained a greater number of SSRs than did those obtained by 5′ sequencing in loblolly pine and spruce [66], the opposite has been observed in *Arabidopsis thaliana* and *Oryza sativa*[67].

**Figure 4:**
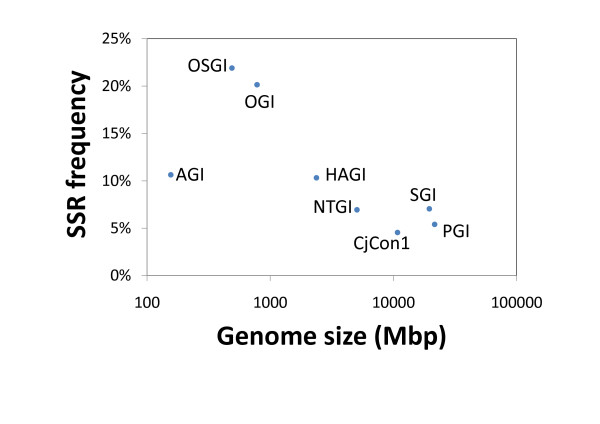
**Relationship between genome size and SSR frequency.** SSR frequencies were plotted against genome size (Mbp) on a log scale. The gene indices are assigned as the following abbreviations: AGI; *Arabidopsis thaliana*, HAGI; *Helianthus annuus*, NTGI; *Nicotiana tabacum*, OGI; Oak, OSGI; *Oryza sativa*, PGI; *Pinus* and SGI; *Picea*. Genome size for *Pinus taeda* and *Picea abies* was used for PGI and SGI, respectively.

A comparison of the SSR frequency in CjCon1 to that in other plant gene indices [41] using the MISA program with a common parameter set revealed that conifer species have relatively low SSR frequencies (5.4, 7.0 and 4.5% for *Pinus* (PGI), spruce (SGI) and *C. japonica* (CjCon1), respectively), whereas SSR frequencies in angiosperms can be as high as 21.9%, as observed in *Oryza sativa* (OSGI). The SSR frequencies (i.e. the ratio of the number of SSR-containing sequences to the total number of sequence in the relevant gene index or CjCon1) were shown to correlate negatively with the logarithm of the genome size (Figure 4) (Spearman’s *r* = −0.81, *P* = 0.022), suggesting that it may be more challenging to develop EST-SSR markers for species with larger genomes such as conifers. Morgante et al. (2002) [65] have previously reported a similar negative correlation between SSR frequency and genome size in plants.

The AT motif was the most common di-SSR in *C. japonica,* accounting for 303 (7.6%) of the 4,003 pure SSRs. However, it is much more common in two other conifer species, accounting for 15.7% and 15.3% of all di-SSRs in *Pinus* (PGI) and spruce (SGI), respectively (Additional file 10: Figure S5). It was also found that penta- and hexa-SSRs are more common in conifers (PGI, SGI and CjCon1) than in the gene indices of other species (Additional file 10: Figure S5). Within these three conifer species, the motif frequencies for PGI and SGI were more strongly correlated than those for CjCon1 and PGI or for CjCon1 and SGI (data not shown). The relatively early split of the Pinaceae (to which *Pinus* and *Picea* belong) from the Cupresseae (to which *Cryptomeria* belongs) probably caused species in the two families to evolve independently [31], which is likely to be reflected in their SSR motif frequencies. However, it should be noted that SSR motif frequencies are not necessarily indicative of phylogenetic relationships [64].

### The location of the EST-SSRs

Estimating the location of microsatellites within genes (coding, 5′ UTR or 3′ UTR) is important when using EST-SSRs to study microsatellite evolution and in marker development. Previous studies have shown that tri-SSRs are preferred in coding regions, because they do not cause frame shift mutations [68] and thus have comparatively few detrimental effects. We sought to determine whether this high frequency of tri-SSRs occurred in CjCon1, and whether the distribution of the SSRs was non-uniform and dependent on their location.

**Figure 5:**
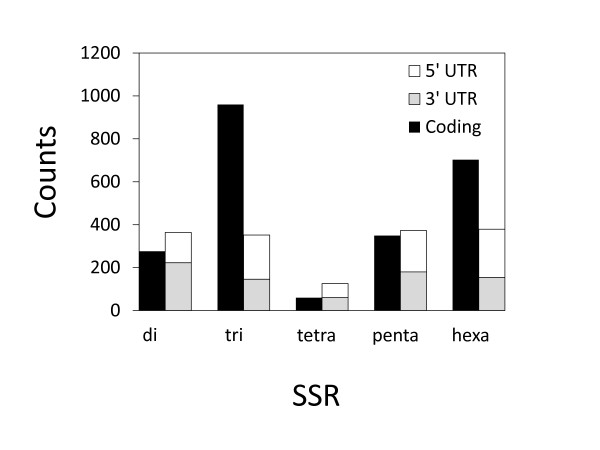
SSR frequency according to estimated location (coding, 3′ UTR or 5′ UTR).

In order to estimate the location of each SSR, the amino acid sequences (i.e. coding regions) of CjCon1 were identified using prot4EST pipeline [44]. The coding sequences were then re-mapped onto the nucleotide sequences to obtain coordinates for the coding regions and estimate the locations of the EST-SSRs. The total length of the estimated coding region was 43.88 Mbp, representing 69.0% of the total sequence length for CjCon1. The average length of the coding region in each contig was 179.9 amino acids (aa), ranging from 14 to 1483 aa. The analysis of coding regions using prot4EST did not guarantee the inclusion of start and/or stop codons (although 28.9% of the identified contigs started with methionine), but rough estimated locations (coding, 5′ UTR or 3′ UTR) were obtained for 3,942 of the 4,059 SSRs; no location could be determined for the remaining 117 because they extended over both estimated coding and non-coding regions. Less than half of the di-SSRs (276 or 42.1%) were estimated to be coding, but 72.8% of the tri-SSRs and 64.3% of the hexa-SSRs were in coding regions (Table 2; Figure 5). Because the addition or deletion of di-SSR repeats located within coding regions can cause frame shifts, selective pressures disfavour the presence of di-SSRs in coding regions [68]. We also examined the locations of specific SSR motifs. The most common motifs in the 3′ UTR and coding regions were AT and AG, respectively, with AT motifs accounting for 154 (23.5%) of all di-SSRs within the 3′ UTRs and AG motifs representing 155 (23.7%) of all di-SSRs motifs in coding regions. AT was also the most common motif in the 5′ UTR, accounting for 70 (10.7%) of all di-SSRs in this region, although it was much less common here than in the 3′ UTR. The most common tri-SSRs in the coding, 3′ UTR, and 5′ UTR regions were AAG, AAT and AGG, respectively.

### GC percentage

Because the genomic GC percentage may affect microsatellite evolution [69], we analyzed the GC content of the contigs, contigs with SSRs, and SSR motifs of CjCon1. The average GC percentage for CjCon1 was 40.23%, with maximum and minimum values of 87.12% and 12.08%, respectively. No homologues of sequences with such extreme GC percentages could be identified using BLAST searches against the NCBI nr database; it is not known whether these sequences are artefacts or represent real transcripts with as-yet unidentified functions. The average GC percentage for the 3,694 SSR-containing contigs was 41.55%, which is greater than that for the entire body of contigs (the difference was significant, as judged by the Welch Two Sample *t*-test; *t* = 15.2978, df = 3968.525, *P* < 2.2e-16). By comparing the GC percentage in CjCon1 to that in other species’ gene indices, it was found that *C. japonica* had the lowest GC percentage of all species examined (Additional file 11: Figure S6). This may be simply because CjCon1 was assembled from both Sanger and pyrosequencing reads, whereas the gene indices were assembled from Sanger reads alone. When assembly was performed using Sanger reads only, the average GC percent of the resulting contigs was 41.42% for *C. japonica*. Because the libraries sequenced by Sanger method were not normalized and the amount of reads was small compared that obtained by pyrosequencing, the resulting transcriptomes were likely to miss genes with low expression, which may have lower GC levels than other genes. We observed a positive relationship between the GC content and the number of reads in contigs (data not shown), which may indicate that highly expressed genes tend to have higher GC contents [70]. When the GC content of contigs containing di- or tri-SSRs was analyzed (the SSR region was masked when calculating the GC content) and related to the GC content of the SSR motifs, a significant positive correlation was observed (Pearson’s *r* = 0.3114, *P* < 2.2 × 10^-16^). Similarly significant correlations were also found for other plant species, with the exception of AGI (*Arabidopsis thaliana*). The lowest and the highest correlations were found for PGI (*Pinus*) (Pearson’s *r* = 0.417) and NTGI (*Nicotiana tabacum*) (Pearson’s *r* = 0.250), respectively.

### Gene ontology

Genic microsatellites have been reported to have functional roles [71], some of which are related to regulatory functions. Tri-SSRs in coding regions generate amino acid repeats whose expansion may cause diseases. We investigated the potential functions of the CjCon1 EST-SSRs by relating them to Gene ontology (GO) annotations. The Blast2GO [50-52] software package was used to assign 97 GO slim terms to 37,387 (46.0%) of the contigs of CjCon1 on the basis of BlastX [47] homology searches against the NCBI nr database. The most frequent GO terms in the Biological process, Cellular component and Molecular function categories were cellular process, intracellular, and binding, respectively (Additional file 12: Table S5). By focusing on contigs with SSRs (of which 1,737 or 47.0% had a GO annotation) and comparing the frequency with which specific GO terms occurred in SSR-containing contigs to the frequency of the same terms in all the contigs of CjCon1, six GO terms were found to be significantly overrepresented in the SSR-containing contigs, with a false discovery rate (FDR) of less than 0.01 (Additional file 12: Table S5). These GO terms included GO:0006351 (Transcription, DNA-dependent), GO:0003677 (DNA binding), GO:0009579 (Thylakoid), GO:0030246 (Carbohydrate binding), GO:0030528 (Transcription regulator activity), GO:0007165 (Signal transduction), GO:0043231 (Intracellular membrane-bounded organelle), in ascending order of FDR. In our previous studies on GO term frequency in EST-SSRs from *Quercus mongolica*[72] and *Castanopsis sieboldii*[73], GO:0003676 (Nucleic acid binding) was found to be overrepresented in EST-SSRs. While GO:0003676 was not found to be significant in this work, GO:0003677 which is located in the lower levels of the GO hierarchy was significantly overrepresented. For *Eucalyptus* EST-SSRs [74], nucleic acid binding was found to be the most common GO term in the Molecular function category. Nucleic acid binding activity is likely to be associated with transcription processes, which were found to be overrepresented in this work. Single amino acid repeats were found to be overrepresented in transcription factors for *Arabidopsis thaliana* and *Oryza sativa* proteins [75].

### The development of EST-SSR markers

We used two pipelines to develop EST-SSR markers, namely read2Marker [15] and CMiB, which combines several freely available tools for clustering and microsatellite detection (see the Methods section and Figure 1 for further details). We used read2Marker scripts to develop EST-SSR markers for several species [72,73,76-79], because of its automated data flow from chromatograms to primer design. Unfortunately, the process used in read2Marker seems to be too inefficient for the huge data sets that are commonly generated using second generation sequencing machines and will become increasingly common in the future. We therefore sought to develop a pipeline that is flexible and capable of efficiently handling the volume of data generated by the second generation sequencers. In order to reduce computational load, we first identified sequences with SSRs. A total of 8,166 SSR-containing sequences were identified, with 2,281 belonging to GroupA, 520 to GroupB, and 5,365 to GroupC, respectively (see the Methods section for details on each group). These SSR-containing sequences were then used for both pipelines.

The first pipeline, read2Marker, was used to design 111 primer pairs, of which 96 were selected to verify the presence of polymorphism. Successful PCR amplification was achieved using 59 (61.5%) of the 96 primer pairs, one of which produced a PCR fragment that was too large (i.e. longer than 600 bp) to be analyzed by a sequencer and was therefore not investigated further. After capillary electrophoresis using a 3100 Genetic Analyzer (Applied Biosystems), 53 primer pairs that produced stable peaks suitable for genotyping were identified; analysis of their products revealed 24 polymorphic loci (Additional file 5: Table S3). For 13 of these, annotation by similarity was possible by comparison with the NCBI nr database (Additional file 13: Table S6). Nineteen of the markers were located within coding regions. The average expected PCR product size for these 24 loci was 240 bp. The number of alleles per locus (*Na*), observed heterozygosity (*H*_*O*_), expected heterozygosity (*H*_*E*_) and *PIC* values were 2–10, 0.06–0.94, 0.06–0.84 and 0.06–0.83, respectively (Additional file 14: Table S7). There was no way to exclude loci corresponding to previously-reported primers [24,26] from the output of read2Marker; as it happened, one of the loci identified (BY909057) had been discussed in a previous study [26].

For the second pipeline, CMiB, we devised a new methodology that emphasises the identification of unique primer pairs that target specific genes and relies on a combination of widely-used programs. This pipeline identified 2,412 primer pairs that were expected to amplify a unique target. This number is substantially greater than that produced by the read2Marker pipeline. Read2Marker uses strict criteria to select specific primers and discards all primer pairs that do not satisfy all criteria, greatly reducing the number of pairs that are ultimately obtained (it should be noted that read2Marker originally identified 2,379 primer pairs using Primer3 [56]). In the CMiB pipeline, mis-annealing between and within sequences was tested for by means of *in silico* PCR experiments using ipcress [58], after which the resulting 2,623 unique PCR products were clustered using BlastCLUST [47] and the primer pairs that produced the shortest products were retained. Because the efficiency of PCR is generally greatest for shorter targets, this approach is likely to yield the greatest possible number of useful candidate primer pairs. After identifying and excluding previously-reported primers [24,26], 96 primer pairs were selected and tested for polymorphism. Fifty-eight (60.4%) of the primer pairs produced PCR products, of which six generated products that were too large (more than 600 bp) to be analyzed by capillary electrophoresis and were therefore discarded. When the PCR products were analyzed by capillary electrophoresis, 41 primer pairs showed clear peak patterns suitable for genotyping. Polymorphisms were detected for 20 loci ( Additional file 5: Table S3), 13 of which could be annotated by similarity with proteins in the NCBI nr database (Additional file 13: Table S6). Fifteen markers targeted coding SSRs. The average expected PCR product size for these 20 loci was 277 bp. The number of alleles per locus (*Na*), observed heterozygosity (*H*_*O*_), expected heterozygosity (*H*_*E*_) and *PIC* values were 2–7, 0.00–0.75, 0.06–0.66 and 0.06–0.60, respectively (Additional file 14: Table S7).

### Factors affecting the PCR success rate and level of polymorphism for EST-SSRs

We used a generalized linear model (GLM) to fit a dependent variable, PCR success/failure (which takes a value of 1 for success and 0 for failure), with four independent variables. Only one of these, the expected PCR product size, was found to have a negative effect on the likelihood of PCR success (Table 3). The other variables, namely the identity of the pipeline used (read2Marker or CMiB) in designing the primers, the location of the primers (i.e. whether the primer pairs were both located in coding regions or not), and the sum of the melting temperatures for the primer pair, had no significant effect on PCR success.

**Table 3:** Factors affecting (a) PCR success and (b) levels of polymorphism, analyzed using generalized linear models

a)				
**Model term**	**Estimate**	**Standard error**	***z* value**	***P***
Pipeline			−0.348	0.728
CMiB	0	0		
read2Marker	−0.1074	0.3090		
Primer location			−0.513	0.6081
coding	0	0		
others	−0.1590	0.3101		
Sum of primer melting temperature	0.1420	0.1191	1.192	0.2333
Expected PCR product size	−0.0033	0.0015	−2.252	0.0244
b)				
**Model term**	**Estimate**	**Standard error**	***z* value**	***P***
Pipeline			0.473	0.636
CMiB	0	0		
read2Marker	0.0727	0.1536		
SSR location			−1.486	0.137
coding	0	0		
others	−0.2824	0.1901		
Maximum No. of SSR repeats	0.1344	0.0233	5.782	7.39E-09
SSR motif (number of SSR repeat unit)	−0.0966	0.1534	−0.63	0.529

PCR success was coded using a variable that took a value of 1 for success and 0 for failure. The primer melting temperature (Tm) was summed for both primers in a pair. The R functions called when estimating PCR success were: glm(formula = PCR.success ~ Pipeline + Primer.location + Sum.of.primer.Tm + Expected.PCR.product.size, family = binomial). The level of polymorphism was expressed in terms of number of alleles per locus (*Na*) and was analyzed using the following function calls in R: glm(formula = Na ~ Pipeline + SSR.location + Maximum.No..of.SSR.repeats + SSR.motif, family = poisson). SSR motif corresponded to the number of bases in the SSR repeat unit; di-, tri-, tetra-, hexa-, and penta-SSRs were coded as 2, 3, 4, 5 and 6, respectively.

We also constructed a GLM fitted with four independent variables to analyze the level of polymorphisms for each primer pair, measured in terms of the number of alleles per locus (*Na*). Only one variable, the maximum number of SSR repeats, had a significant positive effect on *Na* (Table 3). The other three factors considered were the identity of the pipeline used to design the primers, the estimated location of the SSR (i.e. whether it was in a coding or non-coding region), and the nature of the SSR’s repeat unit; none significantly affected the level of polymorphism at the locus. A more detailed examination of the relationship between *Na* and the number of SSR repeats made it possible to identify threshold values for polymorphic EST-SSRs. Di-SSRs with  ≥ 9 repeat units and tri-SSRs with ≥ 10 repeat units all showed polymorphism, while those ≥ 6 and ≥ 4 repeat units, respectively, were polymorphic in some cases. In other words, if seeking to avoid monomorphic markers when designing primers for *C. japonica*, one should target di-SSRs with ≥ 9 repeat units and tri-SSRs with ≥ 10 repeat units. These criteria yielded promising primer pairs for 87 SSRs (3.6% of all SSRs considered) using CMiB. If the aim is to capture *all* polymorphic markers, primers should be designed for di-SSRs with ≥ 6 repeat units and tri-SSRs with ≥ 4. These criteria yielded primer pairs for 1174 SSRs (48.7% of all SSRs considered) using CMiB. Human di-SSR markers exhibit increasing levels of polymorphism as the number of repeat units rises; di-SSRs with more than 10 repeat units were found to be highly informative in a study that examined over 100 markers [80]. It should be noted that the level of polymorphism at a given locus is affected by mutation rates, the characteristics of the species in question (e.g. its mating systems and effective population size, etc.) and the number of samples genotyped. The minimum threshold nucleotide length of polymorphic SSRs has been reported to be 10 bp in humans [81] and 8 in yeasts [82]; these values would correspond to five and four repeat units in di-SSRs, respectively. The identification of threshold lengths for polymorphic SSRs in *C. japonica* will, in conjunction with similar values for other model organisms, facilitate the establishment of criteria for marker development and of critical parameters for the evolutionary analysis of SSRs.

### The genetic diversity of EST-SSR and genomic SSR markers

Forty-two genomic microsatellite markers for *C. japonica* have previously been reported [25]. Their levels of polymorphism (i.e. the number of alleles per locus and *PIC*) were compared to those for the 44 EST-SSR markers identified in the work reported herein. The average values of *Na* and *PIC* were 7.31 and 0.62, respectively, for genomic SSRs; the corresponding values for EST-SSR markers were 3.23 and 0.33. The levels of polymorphism in the genomic SSRs were significantly greater than those for the EST-SSRs (Welch Two Sample *t*-test; *t* = −6.5383, df = 68.278, *P* < 9.388e-9, and *t* = −5.9219, df = 83.822, *P* < 6.777e-8 for *Na* and *PIC*, respectively), probably due to the greater average number of repeats in the genomic SSRs. On average, the maximum number of repeats for all SSR regions was 25.1 for genomic SSRs and 6.0 for EST-SSRs. Genomic SSRs thus had significantly more repeats than EST-SSRs (Welch Two Sample *t*-test; *t* = −6.7532, df = 42.554, *P* < 3.082e-8). The relatively low number of repeats in EST-SSRs could reflect selection against SSR expansion, which generates amino acid repeats in coding regions and affects transcription efficiency in UTRs [71]; both of these effects can cause disease.

## Conclusions

We have developed an open scheme for analysing EST-SSRs and developing EST-SSR markers. All the tools and data used in this scheme are freely available. We collected around 141 k ESTs by Sanger sequencing and 1.3 M ESTs by pyrosequencing from *C. japonica*, an important forestry species in Japan whose genome is significantly smaller than those of other conifers. Assembly using the MIRA program produced 81,284 contigs; 3,694 (4.54%) of these were found to contain SSRs, and 4,059 SSRs were identified in total. The frequency of SSRs was lower than that in seven other species with gene indices. The most common SSRs identified were tri-SSRs; the most common motifs observed were AT and AAG for di- and tri-SSRs, respectively. Tri-SSRs were preferred in coding regions, while di-SSRs were more common in non-coding regions. More SSRs were found in 5′ ESTs than 3′ ESTs. Seven gene ontology terms were found to be overrepresented in the data set. It is expected that further analysis of these characteristic features of EST-SSRs in *C. japonica* will provide useful evolutionary insights.

We developed 44 EST-SSR markers from 192 randomly-selected primer pairs designed using two pipelines, read2Marker [15] and CMiB; the latter of these combines several freely-available tools. Markers resulting from the two pipelines exhibited similar PCR success rates and levels of polymorphism, confirming the utility of the CMiB pipeline.

The analytical scheme and results presented here provide an important foundation for further studies on the genomic and evolutionary analysis of conifers and other non-model species in the age of second generation sequencing.

## Competing interests

The authors declare that they have no competing interests.

## Authors’ contributions

SU conceived this study, analyzed the data and wrote the manuscript. YM prepared DNA samples for EST-SSR analysis and performed genotyping. KU, TUI and NF constructed cDNA libraries. NF and TS constructed full length cDNA libraries and collected EST data. KS and YT coordinated the project. All the authors read and approved the final manuscript.

## Supplementary Material

Additional file 1 **Table S1.** Web links to programs/resources that were used in the present study. Web links were accessed on 19^th^ November 2011. Click here for file

Additional file 2 **Table S2.** Parameters used in Primer3 program for (a) read2Marker and (b) CMiB pipeline. Click here for file

Additional file 3 **cmib.** An example shell script for the CMiB pipeline. Click here for file

Additional file 4 **Figure S1.** Location of *C. japonica* samples used to screen polymorphisms. Click here for file

Additional file 5 **Table S3.** EST-SSR markers for *C. japonica.* Primer sequences are first grouped by pipelines used (read2Marker or CMiB). Forward and reverse primer sequences are listed in upper and lower part of a cell, respectively. Primer sequences include additional bases in 5′ end for fluorescent labelling. Ta: annealing temperature. Click here for file

Additional file 6 **Figure S2.** Length distribution of reads obtained by (A) Sanger and (B) pyrosequencing method. Click here for file

Additional file 7 **Figure S3.** (A) Length and (B) depth distribution of Contigs (CjCon1). Click here for file

Additional file 8 **Table S4.** Assembly statistics from (A) each library, (B) 3′ ESTs and (C) 5′ ESTs. Click here for file

Additional file 9 **Figure S4.** SSR frequency and density for assemblies in different sequencing direction (3′ EST and 5′ EST) for each library. Click here for file

Additional file 10 **Figure S5.** Motif frequency of EST-SSRs in CjCon1 and EST contigs for seven species in gene index. The gene indices are assigned as the following abbreviations: AGI; *Arabidopsis thaliana*, HAGI; *Helianthus annuus*, NTGI; *Nicotiana tabacum*, OGI; Oak, OSGI; *Oryza sativa*, PGI; *Pinus* and SGI; *Picea. * Click here for file

Additional file 11 **Figure S6.** GC percent for CjCon1 and other seven gene indices. The gene indices abbreviations are as follows: AGI; *Arabidopsis thaliana*, HAGI; *Helianthus annuus*, NTGI; *Nicotiana tabacum*, OGI; Oak, OSGI; *Oryza sativa*, PGI; *Pinus* and SGI; *Picea.* Click here for file

Additional file 12 **Table S5.** Summary of gene ontology annotations for CjCon1. Percentage for each GO was based on the total number of sequences with GO annotation. For CjCon1, 37,387 contigs had GO annotation, while for contigs with SSRs, 1,737 had GO annotation. Cells in yellow indicate GOs that are over-represented in SSR-containing ESTs. Click here for file

Additional file 13 **Table S6.** Annotation of EST-SSR markers. #N/A indicates no blast hits. Click here for file

Additional file 14 **Table S7.** Levels of polymorphisms for EST-SSR markers in *C. japonica.* Twenty-four markers from the top are from read2Marker pipeline, while the rest of the markers are from CMiB pipeline. N: number of individuals genotyped; *Na*: number of alleles per locus; *H*_*O*_: observed heterozygosity; *H*_*E*_: expected heterozygosity; *F*_*IS*_: fixation index; *P* value: levels of significance for deviation from Hardy-Weinberg equilibrium; *PIC*: polymorphism information content. Click here for file
