# Supplementary figures and images for: A second generation framework for the analysis of microsatellites in expressed sequence tags and the development of EST-SSR markers for a conifer, Cryptomeria japonica
Source: BMC Genomics. 2012 Apr 16;13:136. doi: 10.1186/1471-2164-13-136 (PMC3424129; doi:10.1186/1471-2164-13-136)

## Slide 1
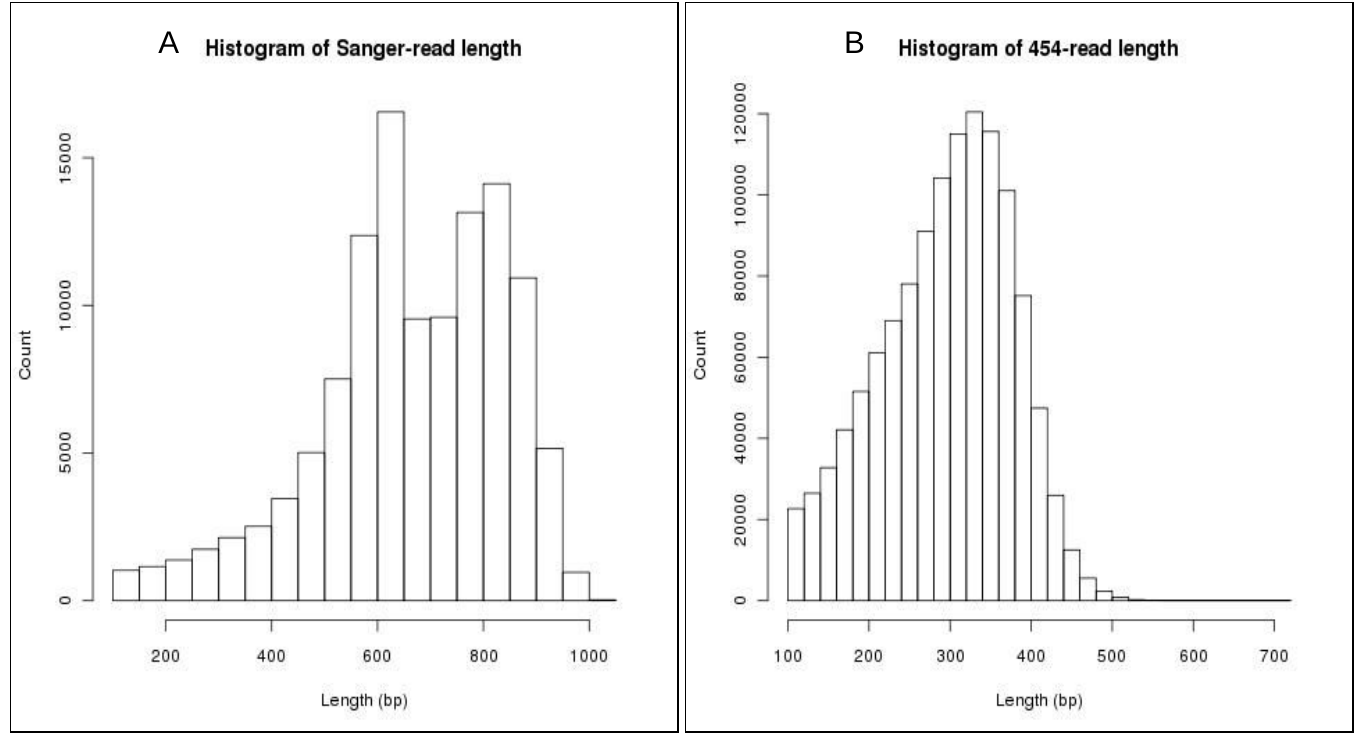

A
B

Supplement: Additional file 1 — Table S1. Web links to programs/resources that were used in the present study. Web links were accessed on 19th November 2011. [file 1471-2164-13-136-S1.ppt]

## Slide 1
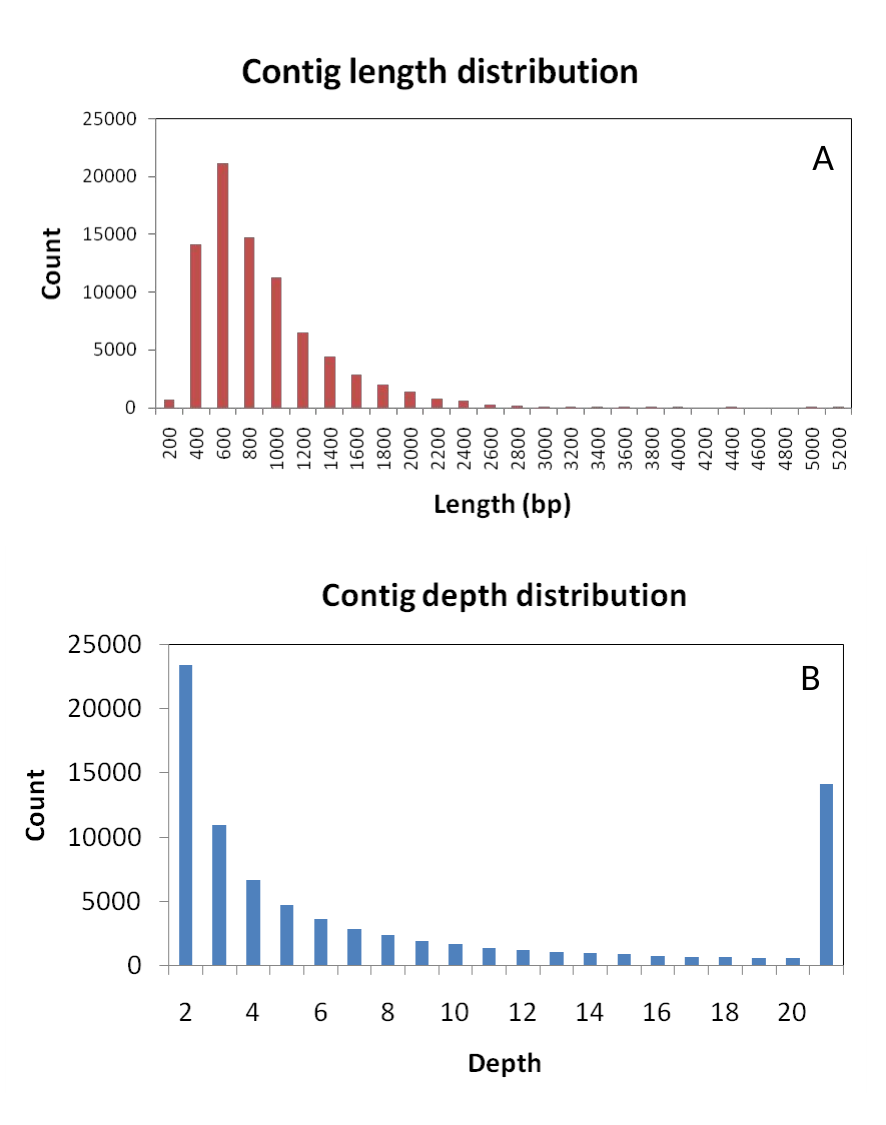

A
B

Supplement: Additional file 2 — Table S2. Parameters used in Primer3 program for (a) read2Marker and (b) CMiB pipeline. [file 1471-2164-13-136-S2.ppt]

## Slide 1
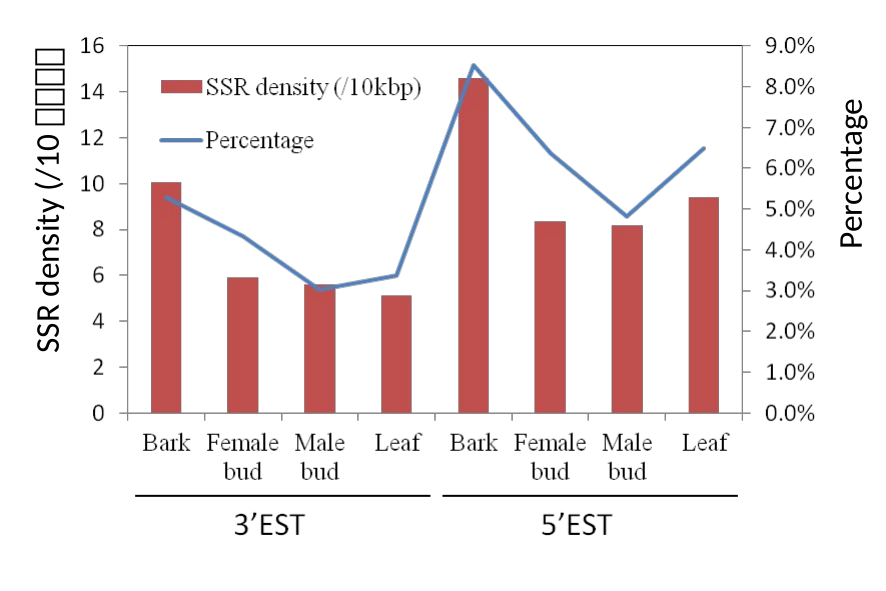

Percentage
SSR density (/10ｋｂｐ）

Supplement: Additional file 4 — Figure S1. Location of C. japonica samples used to screen polymorphisms. [file 1471-2164-13-136-S4.ppt]

## Slide 1
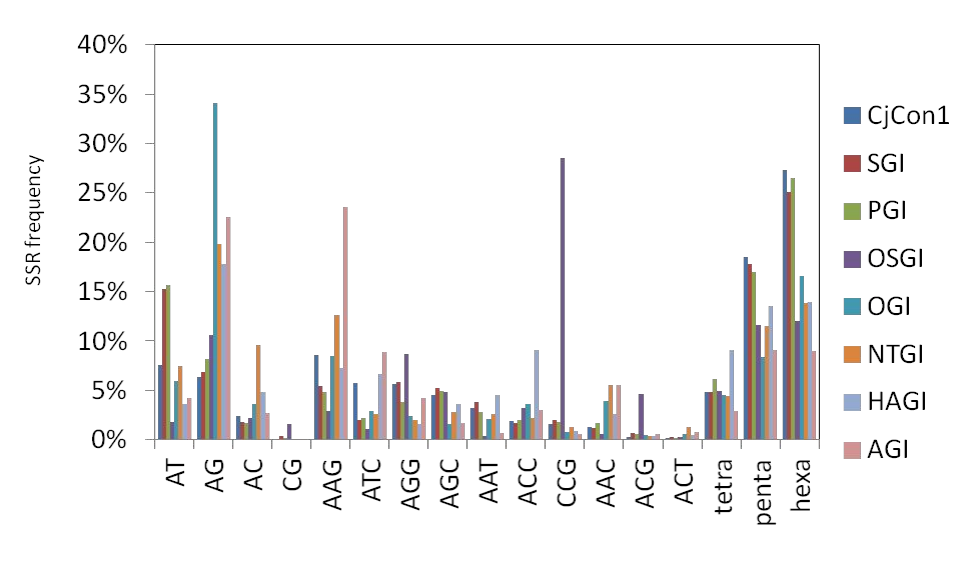

Supplement: Additional file 5 — Table S3. EST-SSR markers for C. japonica. Primer sequences are first grouped by pipelines used (read2Marker or CMiB). Forward and reverse primer sequences are listed in upper and lower part of a cell, respectively. Primer sequences include additional bases in 5′ end for fluorescent labelling. Ta: annealing temperature. [file 1471-2164-13-136-S5.ppt]

## Slide 1
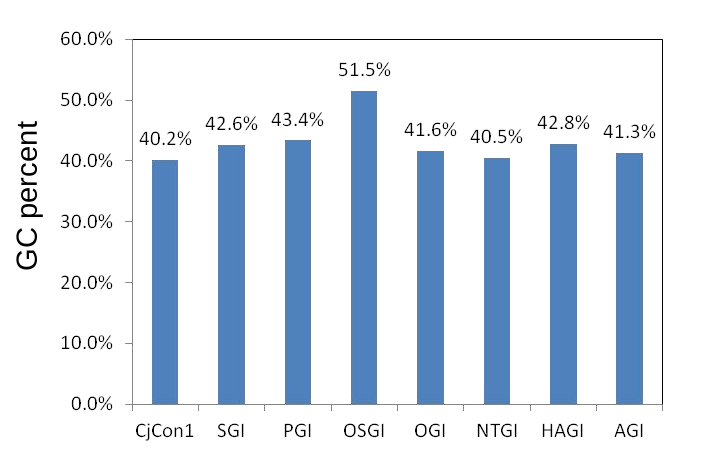

GC percent

Supplement: Additional file 6 — Figure S2. Length distribution of reads obtained by (A) Sanger and (B) pyrosequencing method. [file 1471-2164-13-136-S6.ppt]
