# Supplementary material for: A second generation framework for the analysis of microsatellites in expressed sequence tags and the development of EST-SSR markers for a conifer, Cryptomeria japonica
Source: BMC Genomics. 2012 Apr 16;13:136. doi: 10.1186/1471-2164-13-136 (PMC3424129; doi:10.1186/1471-2164-13-136)
Supplement: Additional file 14 — Table S7. Levels of polymorphisms for EST-SSR markers in C. japonica. Twenty-four markers from the top are from read2Marker pipeline, while the rest of the markers are from CMiB pipeline. N: number of individuals genotyped; Na: number of alleles per locus; HO: observed heterozygosity; HE: expected heterozygosity; FIS: fixation index; P value: levels of significance for deviation from Hardy-Weinberg equilibrium; PIC: polymorphism information content. [file 1471-2164-13-136-S14.ppt]

## Slide 1
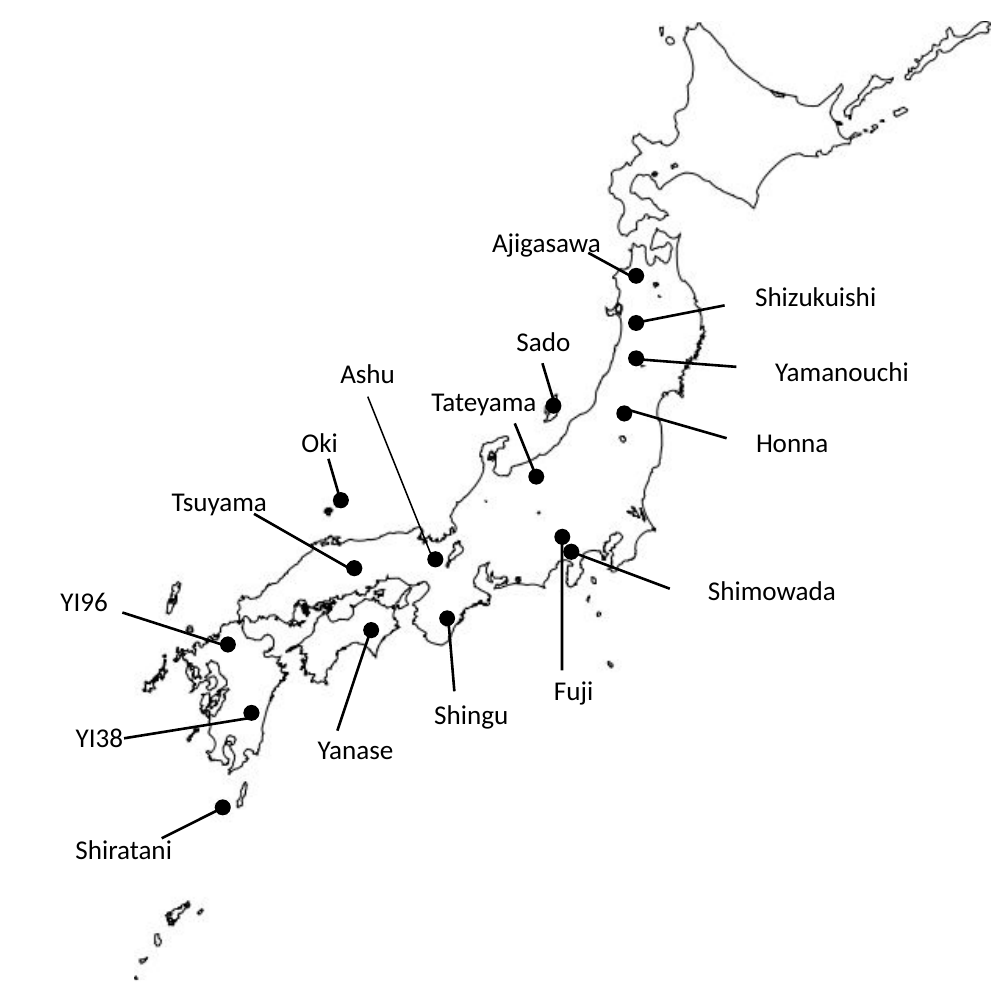

Ajigasawa
Shizukuishi
Sado
Yamanouchi
Tateyama
Oki
Honna
Tsuyama
Shimowada
YI96
Fuji
Shingu
YI38
Yanase
Shiratani
Ashu
